# Body Mass Index and Risk of Gallbladder Cancer: Systematic Review and Meta-Analysis of Observational Studies

**DOI:** 10.3390/nu7105387

**Published:** 2015-09-25

**Authors:** Wenbin Tan, Min Gao, Ning Liu, Guoan Zhang, Tong Xu, Wen Cui

**Affiliations:** 1 Department of Basical Medicine, Jining Medical University, 16 Hehua Road, Jining 272067, China; lzhzsdxsyx@gmail.com; 2 Department of Clinical Laboratory, Jining NO.1 People’s Hospital, 6 Jiankang Road, Jining 272011, China; medchenjun@126.com; 3 Department of Information Technology, Jining Medical University, 16 Hehua Road, Jining 272067, China; liuningsci@126.com; 4 Department of Pathology, Jining Medical University, 16 Hehua Road, Jining 272067, China; zga2007@126.com; 5 Department of Gastrointestinal Surgery, Affiliated Hospital of Jining Medical University, 79 Guhuai Road, Jining 272029, China; ciwinglee@126.com

**Keywords:** overweight, obesity, body mass index, gallbladder cancer, meta-analysis

## Abstract

OBJECTIVES: To provide a quantitative assessment of the association between excess body weight, interpreted as increased body mass index (BMI), and the risk of gallbladder cancer (GBC). METHODS: We identified eligible studies in Medline and EMBASE up to 1 February 2015, and reference lists of retrieved articles. Summary relative risks with their 95% confidence intervals were calculated in a random-effects model. Subgroup analyses were performed according to study design, gender, geographic location, ascertainment of exposure and adjustment for confounders. RESUITS: A total of 12 cohort studies and 8 case-control studies were included in the meta-analysis. Overall, compared with “normal” weight, the summary relative risks of GBC were 1.14 (95% CI, 1.04–1.25) for overweight individuals (BMI 25–30 kg/m^2^) and 1.56 (95% CI, 1.41–1.73) for obese individuals (BMI > 30 kg/m^2^). Obese women had a higher risk of GBC than men did (women: SRRs 1.67, 95% CI 1.38–2.02; men: SRRs 1.42, 95% CI 1.21–1.66), and there was significant association between overweight and GBC risk in women (SRRs 1.26, 95% CI 1.13–1.40), but not in men (SRRs 1.06, 95% CI 0.94–1.20). CONCLUSIONS: Findings from this meta-analysis indicate that obesity is associated with an increased risk of GBC, especially in women. Overweight is associated with GBC risk only in women.

## 1. Introduction

Gallbladder cancer (GBC) is a highly fatal malignancy that differs from other cancers of the biliary tract, as being approximately two to five times more common in women than in men [1]. Prognosis of GBC remains poor due to its late clinical presentation, lack of effective non-operative therapy, and rapid turnover [2].

It has been established that history of gallstone is the leading cause of gallbladder cancer worldwide [3]. Additionally, genetic susceptibility, lifestyle factors, smoking, alcohol consumption and diabetes mellitus (DM) also increase the risk of GBC [4,5,6,7]. Excess body weight, interpreted as overweight (BMI 25–30 kg/m^2^) or obesity (BMI > 30 kg/m^2^), is increasingly recognized as an important risk factor for various cancer types. Over the past decades, evidence from clinical studies has addressed the possible link between excess body weight and risk of GBC, but the findings have been somewhat contradictory. Early studies found no statistically significant results [8,9,10], whereas recent studies did observe a significantly increased risk [11,12].

Our clinical observations indicate a high frequency of obesity among patients with GBC. In the present study, we therefore carried out a systematic review and meta-analysis of all available evidence of observational studies following the meta-analysis of observational studies in epidemiology (MOOSE) guidelines [13] to clarify the association between excess body weight and risk of GBC (Tables S1 and S2).

## 2. Materials and Methods

### 2.1. Search Strategies

Two authors independently performed a literature search using Medline and EMBASE database up to 1 February 2015 with the following text words and/or Medical Subject Heading (MeSH) terms: “body mass index”, “BMI”, “overweight”, “obesity” or “excess body weight”, combined with “gallbladder cancer”, “gallbladder neoplasm” or “biliary tract cancer”. We also reviewed the reference lists of retrieved articles to search for additional studies. No language restrictions were imposed.

### 2.2. Study Selection Criteria

Published articles were included according to the following criteria: (1) the outcome of interest was GBC incidence or mortality; (2) the exposure of interest was overweight or obesity defined by BMI; (3) estimates of odds ratio (OR) or relative risk (RR) with corresponding 95% confidence intervals (CIs) (or data to calculate them) were reported. Two authors independently evaluated all of the studies retrieved from the databases. Any discrepancies between the two reviewers were solved by joint reevaluation of the manuscript. If there were multiple publications from the same study, the most comprehensive one which could provide detail information for subgroup analysis was selected, using other publications to clarify methodology or characteristics of the population.

### 2.3. Data Extraction and Quality Assessment

Three authors independently evaluated all of the studies retrieved according to the aforementioned inclusion criteria. Discrepancies between the three reviewers were solved by a joint reevaluation of the original article. The following information from each included study was extracted: the first author’s last name, geographic location, year of study conducted, sample size, study design, gender and age of participants, duration of follow-up (cohort studies), BMI categories, assessment of BMI (measurement *versus* self-reported), and the effect estimates with 95% CIs. When studies provided more than one RR, we extracted all of them and applied the data according to subgroup analysis. The quality of each study was assessed independently by three reviewers using the Newcastle-Ottawa Scale (NOS). The NOS consists of three parameters of quality: selection, comparability, and outcome (cohort studies) or exposure (case-control studies). The NOS assigns a maximum of four points for selection, a maximum of two points for comparability, and a maximum of three points for exposure or outcome [14]. Any discrepancies between reviewers were addressed by a joint reevaluation of the original article.

### 2.4. Statistical Analysis

To examine associations between overweight/obesity and the risk of GBC, we computed SRRs for two categories of BMI as defined by the World Health Organization (WHO) for adults: overweight (BMI 25–30 kg/m^2^) and obesity (BMI > 30 kg/m^2^ or a discharge diagnosis of obesity) compared with “normal” weight (BMI 18.5–24.9 kg/m^2^). If studies reported relative risk separately for men and women, we combined the gender-specific estimates to the pooled analysis. When non-standard BMI categories were provided, we selected the category that was most closed to those defined by the WHO. Summary relative risk (SRR) estimates with their corresponding 95% CIs were combined in a random-effects model. Subgroup analyses were performed according to study design (cohort and case-control studies), gender (men and women), and geographic location (non-Asia and Asia), BMI assessment (measurement and self-reported), Follow-up time (>10 years and <10 years), smoking status (smokers and non-smokers), Alcohol abuse (Yes and No). We performed sensitivity analysis to estimate the influence of each individual study on the summary results by repeating the random-effects meta-analysis after omitting one study at a time.

To investigate the sources of heterogeneity across these studies, we performed heterogeneity test, and sensitivity analysis. In heterogeneity test, we used the Cochran *Q* and *I*^2^ statistics [15], which were used to test whether the differences found between studies were due to chance. For the *Q* statistic, a *p*-value of less than 0.10 was considered statistically significant heterogeneity. Publication bias was evaluated using funnel plots and the Egger’s test [16]. In the presence of publication bias, we used the “trim and fill” method to correct such bias [17]. Meta-analyses were performed using STATA12.0 (StataCorp., College Station, TX, USA).

## 3. Results

### 3.1. Search Results and Study Characteristics

**Figure 1:**
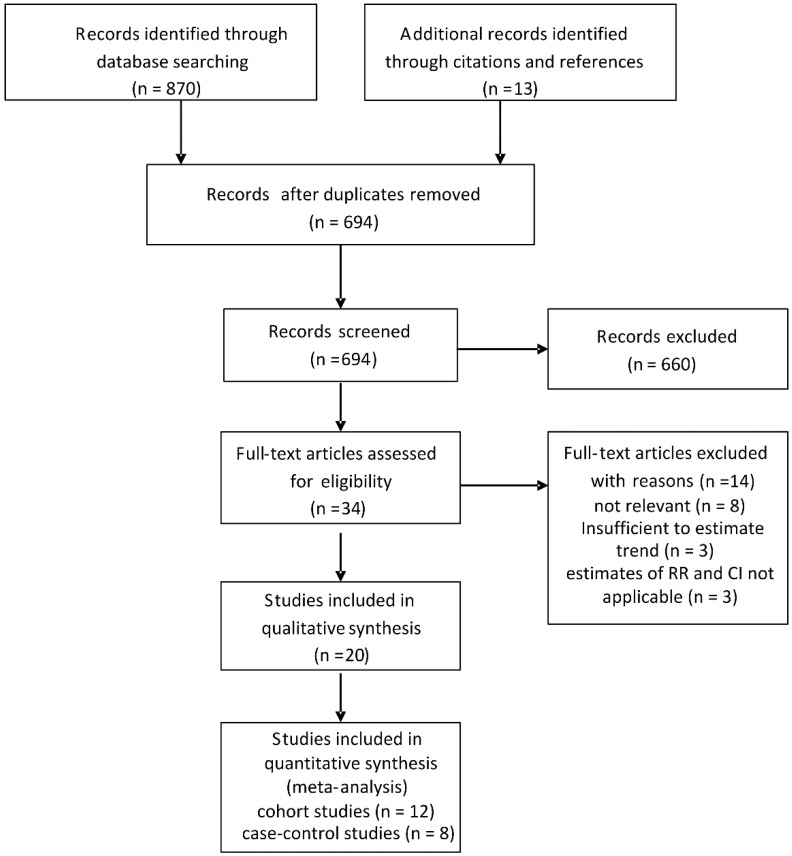
Flow chart of selection of studies included in the meta-analysis.

A total of 883 citations were identified through the literature search. Among the 883 citations, 34 were potentially relevant to the meta-analysis. Among the 34 full text articles, eight studies were not associated with GBC risk, three studies were excluded because gallbladder cancer was not distinguished from extra-hepatic bile duct cancer, and three studies did not provide RR with corresponding CI (or data to calculate them). Finally, a total of 12 cohort studies [8,9,11,18,19,20,21,22,23,24,25,26] (involving 5101 cases) and 8 case-control studies [10,12,27,28,29,30,31,32] (involving 1013 cases and 43,591 controls) with data on BMI and/or obesity related to GBC incidence were included in the meta-analysis (Figure 1). The main characteristics of the included studies were summarized in Table 1 and Table 2. 15 studies were of high quality (NOS ≥ 7). Five studies were of acceptable quality (NOS < 7).

### 3.2. Quantitative Data Synthesis

**Figure 2:**
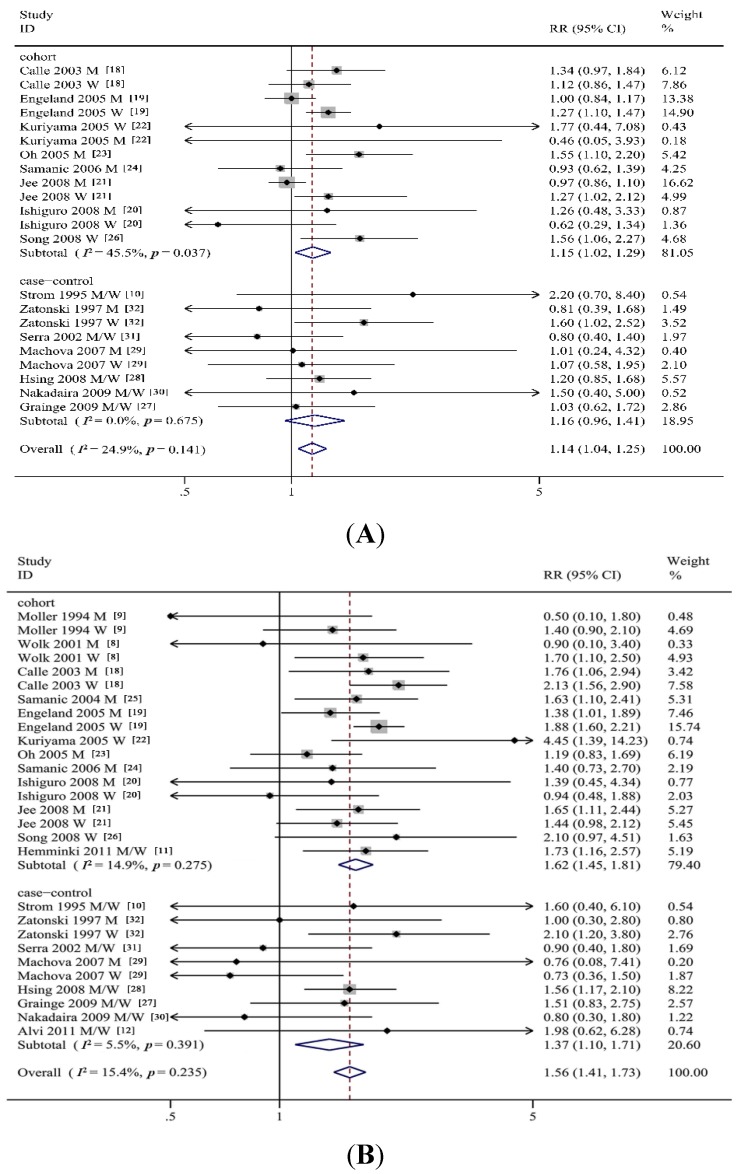
(**A**) Forest plot of risk of gallbladder cancer associated with overweight in general population; (**B**) Forest plot of risk of gallbladder cancer associated with obesity in general population. M, men; W, women; RR, relative risk.

As shown in Figure 2A,B, meta-analysis of the 20 studies in a random-effects model found that a statistically significant positive association was observed between BMI and GBC risk (overweight: SRRs = 1.14, 95% CI = 1.04–1.25, *Q* = 23.85, *I*^2^ = 24.9%; obesity: SRRs = 1.56, 95% CI = 1.41–1.73, *Q* = 37.26, *I*^2^ = 15.4%) compared to “normal weight”. We then performed subgroup analyses by study design, gender, geographic location, ascertainment of exposure and adjustment for confounders, as shown in Table 3.

**Table 1:** Characteristics of eight case-control studies.

Author, Year, Country (Study Period), Source, [Reference No.]	Case Size	Controls Size	Age: Mean or Range	BMI Ascertainment	BMI Categories (kg/m^2^)	Effect Estimate ^a^ (95% CI)		Adjustments	NOS
						Men	Women		
Strom, 1995, Mexico and Bolivia (1984–1988) Hospital, [10]	65	110	45–75	Self-reported	<24.0 24.0–25.9 26.0–28.9 >28.0	Men and women 1.0 (reference) 1.5 (0.5–4.6) 2.2 (0.7–8.4) 1.6 (0.4–6.1)	NA	Age, sex, country	6
Zatonski, 1997, Australia, Canada, The Netherlands and Poland (1983–1988) Population, [32]	189	1479	62.7 m 64.2 f	Self-reported	Quartile 1 Quartile 2 Quartile 3 Quartile 4	1.0 (reference) 1.0 (0.3–3.0) 0.7 (0.3–2.0) 1.0 (0.3–2.8)	1.00 (reference) 1.70 (0.90–3.10) 1.50 (0.80–3.00) 2.10 (1.20–3.80)	Age, sex, center, education, alcohol, smoking, type of interview	7
Serra, 2002, Chile (1992–1995) Hospital, [31]	114	114	65.8 m 70.6 f	Self-reported	<25.0 25.0–29.9 ≥30.0	Men and women 1.0 (reference) 0.8 (0.4–1.4) 0.9 (0.4–1.8)	NA	Age, sex	7
Máchová, 2007, Czech (1987–2002) Population, [29]	93	37772	30–64	Measured	18.5–24.9 25–30 ≥30.0	1.00 (reference) 1.01 (0.24–4.32) 0.76 (0.08–7.41)	1.00 (reference) 1.07 (0.58–1.95) 0.73 (0.36–1.50)	Age, smoking, height, hypertension	8
Hsing, 2008, China (1997–2001) Population, [28]	365	959	34–74	Self-reported	<18.5 18.5–22.9 23.0–24.9 ≥25	Men and women 0.62 (0.35–1.09) 1.0 (reference) 1.2 (0.85–1.68) 1.56 (1.17–2.10)	NA	Age, sex, education	6
Grainge, 2009, United Kingdom (1987–2002) Population, [27]	86	3007	72	Measured	<25 25–29.9 ≥30.0	Men and women 1.00 (reference) 1.03 (0.62–1.72) 1.51 (0.83–2.75)	NA	Smoking, alcohol, NSAID use	8
Nakadaira, 2009, Hungary (2003–2006) hospital, [30]	41	30	40–69	NA	≤24.9 25.0–29.9 ≥30.0	Men and women 1.00 (reference) 1.5 (0.4-5.0) 0.8 (0.3–1.8)	NA	Age	7
Alvi, 2011, Pakistan (1988–2007) hospital, [12]	60	120	18–75	Measured	<23 >23	Men and women 1.00 (reference) 1.98 (0.62–6.28)	NA	Sex, hypertension, diabetes, smoking	7

NA data not applicable; m, male; f, female; ^a^ relative risks are rate ratios, odds ratios, or standardized incidence ratios.

**Table 2:** Characteristics of 12 cohort studies.

Author, Year, Country, (Study Period) [Ref. No.]	Total Cohort	Age: Mean or Range	Cases	Follow-up, Years	BMI Ascertainment	BMI Categories (kg/m^2^)	Effect Estimate ^a^ (95% CI)		Adjustments	NOS
							Men	Women		
Moller, 1994, Denmark (1977–1987), [9]	43965	50 m 60 f	28	5	Discharge diagnosis	Non-obese Obese	1.00 (reference) 0.50 (0.1–1.8)	1.00 (reference) 1.40 (0.9–2.1)	Age	6
Wolk, 2001, Sweden (1965–1993), [8]	28129	46.1	31	10.3	Discharge diagnosis	Non-obese Obese	1.00 (reference) 0.90 (0.1–3.4)	1.00 (reference) 1.70 (1.1–2.5)	Age, calendar year	7
Calle, 2003, United States (1982–1998), [18]	900053	57	484	16	Self-reported	18.5–24.9 25.0–29.9 30.0–34.9	1.00 (reference) 1.34 (0.97–1.84) 1.76 (1.06–2.94)	1.00 (reference) 1.12 (0.86–1.47) 2.13 (1.56–2.90)	Age, race, marital status, smoking, aspirin, alcohol, estrogen therapy (w)	8
Samanic, 2004, United States (1969–1996), [25]	4500700 m	52.18 whites 47.63 blacks	338 m	12	Discharge diagnosis	Non-obese Obese	1.00 (reference) 1.63(1.10–2.41) ^b^	NA	Age, calendar year	6
Engeland, 2005, Norway (1963–2001), [19]	2001719	20–74	1,715	13	Measured	18.5–24.9 25.0–29.9 ≥30.0	1.00 (reference) 1.00 (0.84–1.17) 1.38 (1.01–1.89)	1.00 (reference) 1.27 (1.10–1.47) 1.88 (1.60–2.21)	Age, birth cohort	7
Kuriyama, 2005, Japan (1984–1992), [22]	27539	≥40	33	9	Self-reported	18.5–24.9 25.0–27.4 27.5–29.9 ≥30.0	1.00 (reference) 0.46 (0.05–3.93)	1.00 (reference) 0.83 (0.23–2.98) 3.43 (1.19–9.94) 4.45(1.39–14.23)	Age, smoking, health insurance, alcohol	7
Oh, 2005, Korea (1992–2001), [23]	781283 m	≥20	182	10	Measured	21.0–22.9 23.0–24.9 25.0–26.9 27.0–29.9	1.00 (reference) 1.55 (1.10–2.20) 1.15 (0.74–1.80) 1.25 (0.70–2.24)	NA	Age, smoking, alcohol, exercise, region	7
Samanic, 2006, Sweden (1971–1999), [24]	362552 m	34.3	109	19	Measured	18.5–24.9 25.0–29.9 ≥30.0	1.0 (reference) 0.93 (0.62–1.39) 1.40 (0.73–2.70)	NA	Age, smoking	8
Ishiguro, 2008, Japan (1994–2004), [20]	101868	40–69	90	10.9	Self-reported	≤22.9 23.0–24.9 25.0–26.9 ≥27.0	1.00 (reference) 0.74 (0.28–1.92) 1.26 (0.48–3.33) 1.39 (0.45–4.34)	1.00 (reference) 0.47 (0.22–0.98) 0.62 (0.29–1.34) 0.94 (0.48–1.88)	Age, gender, study area, diabetes, smoking, alcohol	6
Jee, 2008, Korean (1992–2006), [21]	1213829	45.0 m 49.4 f	1882	10.8	Measured	23.0–24.9 25.0–29.9 ≥30	1.00 (reference) 0.97 (0.86–1.10) 1.65 (1.11–2.44)	1.00 (reference) 1.27 (1.02–2.12) 1.44 (0.98–2.12)	Age, smoking	8
Song, 2008, Korean (1994–2003), [26]	170481 f	55.9	181	8.75	Measured	21.0–22.9 23.0–24.9 25.0–26.9 27.0–29.9 ≥30	NA	1.00 (reference) 1.06 (0.62–1.80) 1.30 (0.76–2.22) 1.86 (1.09–3.18) 2.10 (0.97–4.51)	Age, height, smoking, alcohol, exercise, pay level	7
Hemminki, 2011, Sweden (1964–2006), (11)	30020	NA	28	11.2	Discharge diagnosis	Non-obese obese	Men and women 1.00 (reference) 1.73 (1.16–2.57)^c^	NA	Age, sex, region, economic status	7

NA data not applicable; m, male; f, female; ^a^ relative risks are rate ratios, odds ratios, or standardized incidence ratios; ^b^ combined whites and blacks; ^c^ combined obesity and family obesity.

**Table 3:** Subgroup analysis of relative risks for association between body mass index (BMI) and gallbladder cancer risk.

	Overweight					Obesity				
	Studies, *n*	RR (95% CI)	*p*_h_	*Q*	*I*^2^,%	Studies, *n*	RR (95% CI)	*p*_h_	*Q*	*I*^2^,%
**Study design**										
Cohort studies	12	1.15 (1.02–1.29)	0.04	22.03	45.5	12	1.62 (1.45–1.81)	0.28	19.98	14.9
Case-control studies	8	1.16 (0.96–1.41)	0.68	5.75	0	8	1.37 (1.10–1.71)	0.39	9.52	5.5
**Follow-up time**										
>10 years	6	1.12 (1.00–1.27)	0.04	17.78	49.4	9	1.65 (1.49–1.83)	0.40	13.58	4.3
<10 years	2	1.52 (1.06–2.19)	0.54	1.22	0	3	1.69 (0.91–3.17)	0.10	6.32	52.5
**Control source**										
Hospital	3	1.14 (0.61–2.03)	0.30	2.39	16.4	4	1.07 (0.66–1.74)	0.57	2.03	0
Population	4	1.18 (0.96–1.46)	0.67	3.19	0	4	1.43 (1.09–1.89)	0.30	6.12	18.3
**Sex**										
Men	9	1.06 (0.94–1.20)	0.24	10.33	22.5	11	1.42 (1.21–1.66)	0.85	5.63	0
Women	8	1.26 (1.13–1.40)	0.45	6.84	0	10	1.67 (1.38–2.02)	0.06	16.38	45.0
**Geographic region**										
Asia	6	1.19 (0.98–1.45)	0.06	15.00	46.7	7	1.48 (1.26–1.74)	0.43	8.07	0.9
Non-Asia	9	1.14 (1.05–1.25)	0.43	12.20	1.7	13	1.58 (1.40–1.80)	0.22	22.38	19.6
**BMI ascertainment**										
Self-reported	7	1.18 (1.01–1.36)	0.46	9.74	0	7	1.65 (1.32–2.05)	0.20	12.16	26.0
Measured	7	1.14 (1.01–1.30)	0.04	17.40	48.3	8	1.51 (1.29–1.77)	0.20	13.52	26.1
**Adjustment for confounders smoking**										
Yes	10	1.16 (1.02–1.31)	0.16	20.42	26.5	11	1.55 (1.31–1.83)	0.21	19.06	21.3
No	5	1.14 (0.98–1.32)	0.21	7.10	29.5	9	1.59 (1.40–1.80)	0.32	12.61	12.8
**Alcohol intake**										
Yes	7	1.27 (1.10–1.47)	0.37	10.87	8.0	7	1.64 (1.31–2.07)	0.15	13.31	32.4
No	8	1.08 (0.98–1.19)	0.25	12.59	20.6	13	1.56 (1.40–1.73)	0.36	18.44	7.8

RR, relative risk; CI, confidence interval; BMI, body mass index; *p*_h_, *p*-value for heterogeneity; *Q*, Cochran’s Q statistics.

In stratified analysis by study design, a statistically significant positive association between BMI and GBC risk was observed for the cohort studies (overweight: SRRs = 1.15, 95% CI = 1.02–1.29; obesity: SRRs = 1.62, 95% CI = 1.45–1.81). Moreover, in cohort studies with follow-up time >10 years, overweight and obesity were strongly associated with incidence of GBC (overweight: SRRs = 1.12, 95% CI = 1.00–1.27; obesity: SRRs = 1.65, 95% CI = 1.49–1.83), while only overweight was observed associated with GBC risk in cohort studies with follow-up time < 10 years (SRRs = 1.52, 95% CI = 1.06–2.19). For the case-control studies, only obesity was strongly associated with GBC risk (SRRs = 1.37, 95% CI = 1.10–1.71). The SRRs of GBC incidence for obesity in population-based case-control studies was 1.43 (95% CI = 1.09–1.89); no significant association between obesity and GBC risk was observed in hospital-based case-control studies.

A significant gender-specific difference was observed in the association between obesity and GBC risk, and obese women had a higher risk of GBC (women: SRRs = 1.67, 95% CI = 1.38–2.02; men: SRRs = 1.42, 95% CI = 1.21–1.66). However, overweight men are not associated with risk of GBC (SRRs = 1.06, 95% CI = 0.94–1.20).

In stratified analysis by geographic location, the association between obesity and the risk of GBC was similar for both Asia and non-Asia (Table 3). For non-Asians, overweight was strongly associated with GBC incidence (SRRs = 1.14, 95% CI = 1.05–1.25; no significant association between overweight and the GBC risk was observed for Asians. In stratified analysis by BMI ascertainment, both overweight and obesity had a higher risk of GBC in self-report studies

In addition, when stratified by potential confounders, overweight people with smoking and alcohol consumption were strongly associated GBC risk, no significant association between overweight and the risk of GBC was found in those without smoking and alcohol consumption (non-smokers: SRRs = 1.14, 95% CI = 0.98–1.32; non-alcoholics: SRRs = 1.08, 95% CI = 0.98–1.19), indicating that smoking and alcohol consumption are positive confounders. No differences were observed in the association between obesity and GBC incidence when stratified by smoking and alcohol consumption.

### 3.3. Sensitivity Analyses and Publication Bias

In the sensitivity analyses, we removed one study at a time and calculated the SRRs. We found that there were no changes in the direction of effect when any one study was excluded, supporting the robustness of our results. For example, when the study of Engeland *et al.* [19] was excluded (which seemed to have a strong influence on the estimate of effect), the SRR remained similar with the overall pooled RRs (SRRs = 1.15, 95% CI = 1.03–1.28, *I*^2^ = 17.8%).

**Figure 3:**
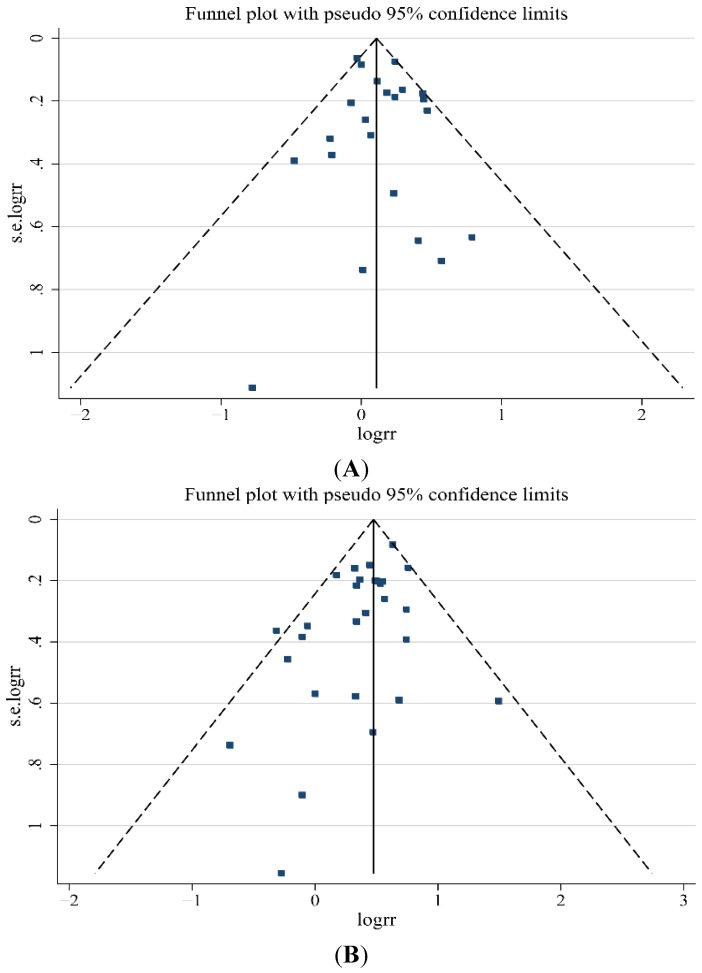
(**A**) Funnel plot of studies evaluating the association between overweight and gallbladder cancer risk (*p* = 0.483); (**B**) Funnel plot of studies evaluating the association between obesity and gallbladder cancer risk (*p* = 0.019).

No indication of publication bias was observed in the literature on BMI and GBC risk in overweight group based on the Egger’s test (*p* = 0.483) results (Figure 3A). For BMI and GBC risk in the obesity group, the funnel plot showed a little asymmetry (Figure 3B), indicating some evidence of bias. However, when the “trim and fill” approach was performed, data was unchanged, suggesting that the effect of publication bias could be negligible.

## 4. Discussion and Conclusions

In this meta-analysis, we found that overweight and obesity were associated with 14% and 56% excess risk of GBC, respectively. Our results are consistent with previous studies that the association between obesity and GBC risk was higher in women than men. Women with overweight had a higher risk of GBC, while no significant association between overweight and the GBC risk was observed for men.

Several biological mechanisms were hypothesized for the possible carcinogenesis of gallbladder associated with excess body weight. Obesity is often accompanied with metabolic syndrome, characterized by insulin resistance, hyperglycemia, dyslipidemias, and hypertension [33]. In obese adults, alterations occur in the circulating levels of insulin, insulin-like growth factor (IGF)-1, adipokines, inflammatory factors, and pro-inflammatory cytokines. These mediators associated with the obesity, contribute to cancer-related processes, including growth signaling, inflammation, and vascular alterations [34]. Furthermore, obesity and metabolic syndrome are risk factors for gallstone disease [35], which may indirectly increase the risk of GBC [36]. In addition, female sex hormones adversely influence hepatic bile secretion and gallbladder function [37]. Estrogens increase cholesterol secretion and diminish bile salt secretion, while progestins act by reducing bile salt secretion and impairing gallbladder emptying leading to stasis [38]. These may partially explain the stronger association observed with overweight or obesity in women than in men.

Our meta-analysis has several strengths. (1) This meta-analysis was based on 20 epidemiologic studies, which might minimize the possibility of selection bias. (2) Most of the included studies provided more than one RRs, which could be applicable to accurately subgroup analysis. (3) The included studies evaluated multiple confounders including smoking and alcohol. The relationships between BMI and risk of GBC in each study were derived from regression after adjustment at least for age and gender.

Our meta-analysis has limitations that affect interpretation of the true results. First, inadequate control for confounders may bias the results, leading to exaggeration or underestimation of risk estimates. Thus, when interpreting the association between excess body weight and GBC risk, possible unmeasured or residual confounding factors should be considered. Five studies were of acceptable quality (NOS < 7), mainly due to the adjustments made for confounders. Smoking and alcohol abuse is closely related to GBC risk. Subgroup analysis results also shown that overweight people with smoking and alcohol consumption were strongly associated GBC risk, while no significant association between overweight and the risk of GBC was found in those without smoking and alcohol consumption, suggesting that data from unadjusted studies might lead to an overestimation of overweight in the development of GBC. Interestingly, no differences were observed in the association between obesity and GBC incidence when stratified by smoking and alcohol consumption, suggesting that obesity might be an independent risk factor. Gallstone is closely related to GBC risk [39]. Meanwhile, obesity tends to be accompanied with DM, which is also associated with increased GBC risk [4,40]. However, most studies did not adjust for these risk factors. This could have led to an overestimation of the true association between obesity and risk of GBC. Second, although BMI is the most commonly used anthropometric tool to assess relative weight and classify obesity, BMI cannot make the distinction between an excess body weight due to high levels of fat mass or muscle mass. Generally, an excess fat mass is more frequently associated with metabolic syndrome than a high level of muscle mass, leading to increased risk of GBC. Furthermore, obese individuals differ in regional body fat distribution. Adipose tissue now is considered as an endocrine organ, playing an important role in tumor microenvironment. Abdominal adiposity might play a more important role than peripheral type of obesity in the development of abdominal cancer. Other tools, such as waist circumference (WC), waist-to-height ratio (WHtR), and waist-to-hip ratio (WHR), which are more useful than BMI in determining abdominal adiposity, might be more sensitive in predicting the risk of abdominal cancer. However, little clinical evidence can be achieved to compare the screening potential of each tool. Third, several studies in this meta-analysis relied on self-reported weight and height data, which may attenuate the relative risk estimates. However, the SRRs for BMI ascertained by measurement were similar to those by self-reported. Finally, as in any meta-analysis, the possibility of publication bias is of concern, because a few studies with null results tend not to be published. However, the results from this study did not provide evidence for such a bias.

There was significant heterogeneity observed across studies about overweight and GBC risk, but the heterogeneity is low and acceptable with *I*^2^ = 31.9%, so we could combine studies in a meta-analysis. We analyzed this review in both fixed effects and random effects, and found that they had no significant differences. Thus, the more conservative one, random effects, was chosen finally. Next, when we tried to carry out subgroup analysis to investigate sources of heterogeneity, statistical heterogeneity was lower in analysis of case-control studies, population based studies, Non-Asia studies and studies of BMI ascertainment by self-report, indicating that these might account for heterogeneity observed in studies about overweight and GBC risk.

In summary, findings of this meta-analysis provide evidence that excess body weight may increase GBC risk. Further studies that meet strict criteria on this subject are needed to strengthen the association between BMI and GBC risk, especially those adjusting potential confounding factors such as gallstones and DM.

## Supplementary Files

Supplementary File 1

## Abbreviations

GBC: Gallbladder cancer
BMI: Body mass index
OR: Odds ratio
RR: Relative risk
HR: Hazard ratio
SRRs: Summary relative risks
CI: Confidence intervals
DM: Diabetes mellitus
