# Supplementary material for: Body Mass Index and Risk of Gallbladder Cancer: Systematic Review and Meta-Analysis of Observational Studies
Source: Nutrients. 2015 Sep 25;7(10):8321–34. doi: 10.3390/nu7105387 (PMC4632410; doi:10.3390/nu7105387)
Supplement: Supplementary File 1 [file nutrients-07-05387-s001.docx]

Supplementary Materials

Table S1. MOOSE Checklist.

| **Criteria** | | **Brief description of how the criteria  were handled in the meta-analysis** |
| --- | --- | --- |
| **Reporting of background should include** | |  |
| √ | Problem definition | Gallbladder cancer (GBC) is a highly fatal malignancy that differs from other cancers of the biliary tract. Excess body weight, interpreted as overweight (BMI 25-30 kg/m^2^) or obesity (BMI > 30 kg/m^2^), is increasingly recognized as an important risk factor for various cancer types. So many studies have addressed the possible link between excess body weight and risk of GBC, but the findings have been somewhat contradictory. We therefore carried out a meta-analysis to clarify the association between excess body weight and risk of GBC. |
| √ | Hypothesis statement | Overweight and obesity increases the risk of GBC |
| √ | Description of study outcomes | Incidence of GBC |
| √ | Type of exposure or intervention used | Overweight or obesity defined by BMI |
| √ | Type of study designs used | Using a case-control, cross-sectional, nested case-control, or cohort design. |
| √ | Study population | We placed no restriction. |
| **Reporting of search strategy should include** | |  |
| √ | Qualifications of searchers | The credentials of the three investigators Y.G, K.S and C.M are indicated in the author list. |
| √ | Search strategy, including time period included in the synthesis and keywords | PubMed up to 1 February 2013  EMBASE up to 1 February 2013  Text words and/or Medical Subject Heading (MeSH) terms: “body mass index”, “BMI”, “overweight”, “obesity” or “excess body weight”, combined with “gallbladder cancer”, “gallbladder neoplasm” or “biliary tract cancer” |
| √ | Databases and registries searched | PubMed and EMBASE |
| √ | Search software used, name and version, including special features | We did not employ a search software. NoteExpress was used to merge retrieved citations and eliminate duplications |
| √ | Use of hand searching | We hand-searched bibliographies of retrieved papers for additional references |
| √ | List of citations located and those excluded, including justifications | Details of the literature search process are outlined in the flow chart. The citation list is available upon request |
| √ | Method of addressing articles published in languages other than English | No language restrictions were imposed, however, all included studies were published in English. |
| √ | Method of handling abstracts and unpublished studies | Unpublished studies were excluded in the meta-analysis. |
| √ | Description of any contact with authors | We did not contact authors for the detailed information of primary studies. RR with corresponding 95% CI was available or could be calculated from raw data. |

**Table S1.** *Cont*.

| **Criteria** | | **Brief description of how the criteria  were handled in the meta-analysis** |
| --- | --- | --- |
| **Reporting of methods should include** | |  |
| √ | Description of relevance or appropriateness of studies assembled for assessing the hypothesis to be tested | Detailed inclusion and exclusion criteria were described in the methods section. |
| √ | Rationale for the selection and coding of data | Data extracted from each of the studies were relevant to the population characteristics, study design, exposure, outcome, and possible effect modifiers of the association. |
| √ | Assessment of confounding | Subgroup analyses were carried out by study design, gender, geographic location, BMI assessment, follow-up time, smoking status, and alcohol abuse. In the sensitivity analyses, we removed one study at a time and calculating the SRRs. |
| √ | Assessment of study quality, including blinding of quality assessors; stratification or regression on possible predictors of study results | The quality of each study was assessed independently by three reviewers using the Newcastle - Ottawa Scale (NOS). |
| √ | Assessment of heterogeneity | In heterogeneity tests, we used the Cochran *Q* and *I*^2^ statistics, which were used to test the differences obtained between studies due to chance. For the *Q* statistic, a *p*-value of less than 0.10 was considered representative of statistically significant heterogeneity. We also tried to carry out subgroup analysis to investigate sources of heterogeneity. |
| √ | Description of statistical methods in sufficient detail to be replicated | Description of methods of meta-analyses, heterogeneity tests, sensitivity analyses, and assessment of publication bias are detailed in the methods. |
| √ | Provision of appropriate tables and graphics | We included one flow chart, two forest plots and two funnel plots for overweight and obesity, respectively, two summary tables of the characteristics of included studies, and one table for subgroup analyses. |
| **Reporting of results should include** | |  |
| √ | Graph summarizing individual study estimates and overall estimate | Figure 2A and 2B |
| √ | Table giving descriptive information for each study included | Table 1 and Table 2 |
| √ | Results of sensitivity testing | Page 7 Line 19 sensitivity analyses and publication bias |
| √ | Indication of statistical uncertainty of findings | 95% confidence intervals were presented with all summary estimates, *I*^2^ values and results of sensitivity analyses. |

**Table S1.** *Cont*.

| **Criteria** | | **Brief description of how the criteria  were handled in the meta-analysis** |
| --- | --- | --- |
| **Reporting of discussion should include** | |  |
| √ | Quantitative assessment of bias | Sensitivity analyses indicate heterogeneity in strengths of the association due to most common biases in observational studies. |
| √ | Justification for exclusion | We excluded studies that had not adjusted for or were standardized by age, a potential confounder, and used different exposure or outcome assessment for the comparison groups. Those studies that did not report RR and corresponding CI  (or data to calculate them). |
| √ | Assessment of quality of included studies | We discussed the strengths of this meta-analysis and potential reasons for the observed heterogeneity. |
| **Reporting of conclusions should include** | |  |
| √ | Consideration of alternative explanations for observed results | We discussed that potential unmeasured confounders such as gallstone and DM may have caused residual confounding. This could have led to an overestimation of the true association between obesity and risk of GBC.  We also noted that the self-reported weight and height data may attenuate the relative risk estimates. |
| √ | Generalization of the conclusions | Our conclusion was not generalizable due to unmeasured confounders which have a substantial impact on SRRs. The definition overweight/obesity by BMI is tiny different across countries, and we use the definition of WHO for adults. We noted the lack of studies in Africa. |
| √ | Guidelines for future research | We recommend future studies that meet epidemiologic criteria on this subject are needed to strengthen the association between BMI and GBC risk, especially those adjusting potential confounding factors such as gallstones and DM. |
| √ | Disclosure of funding source | This work was supported by grants from the National Natural Science Foundation of China (No. 81271876), Natural Science Foundation of Shandong Province (No. ZR2011HL004), Jining Science and Technology Project (No. 2014JNNK21-Liu Ning, 2010-Tan Wenbin )，Program for Innovation of Graduate Education of Shandong Province (No. SDYY14014). |

**Table S2.** PRISMA Checklist.

| **Section/topic** | **#** | **Checklist item** | **Reported on page #** |
| --- | --- | --- | --- |
| **TITLE** | | |  |
| Title | 1 | Body mass index and risk of Gallbladder cancer: systematic review and meta-analysis of observational studies | 1 |
| **ABSTRACT** | | |  |
| Structured summary | 2 | OBJECTIVES: To provide a quantitative assessment of the association between excess body weight, interpreted as increased body mass index (BMI), and the risk of gallbladder cancer (GBC).  METHODS: We identified eligible studies in Medline and EMBASE up to 1 February 2013, and the reference lists of retrieved articles. Summary relative risks with their 95% confidence intervals were calculated with a random-effects model.  RESUITS: A total of 12 cohort studies and 8 case-control studies were included in the meta-analysis. Overall, compared with “normal” weight, the summary relative risks of GBC were 1.14 (95% CI, 1.04–1.25) for overweight individuals (BMI  25–30 kg/m2) and 1.56 (95% CI, 1.41–1.73) for obese individuals (BMI > 30 kg/m^2^). Obese women had a higher risk of GBC than men did (women: SRRs 1.67, 95% CI 1.38–2.02, men: SRRs 1.42, 95% CI 1.21–1.66), and there was a significant association between overweight and GBC risk for women (SRRs 1.26, 95% CI 1.13–1.40), but not for men (SRRs 1.06, 95% CI 0.94–1.20).  CONCLUSIONS: Findings from this meta-analysis indicate that obesity is associated with an increased risk of GBC, especially in women. Overweight is associated with GBC risk only in women. | 2 |
| **INTRODUCTION** | | |  |
| Rationale | 3 | Gallbladder cancer (GBC) is a highly fatal malignancy that differs from other cancers of the biliary tract. Excess body weight, interpreted as overweight (BMI 25–30 kg/m^2^) or obesity (BMI > 30 kg/m^2^), is increasingly recognized as an important risk factor for various cancer types. So many studies have addressed the possible link between excess body weight and risk of GBC, but the findings have been somewhat contradictory. | 3 |
| Objectives | 4 | We carried out a systematic review and meta-analysis of all available evidence of observational studies to clarify the association between excess body weight and risk of GBC. | 3 |

**Table S2.** *Cont.*

| **Section/topic** | **#** | **Checklist item** | **Reported on page #** |
| --- | --- | --- | --- |
| **METHODS** | | |  |
| Protocol and registration | 5 | Following the meta-analysis of observational studies in epidemiology (MOOSE) guidelines. | 3 |
| Eligibility criteria | 6 | (1) the outcome of interest was GBC incidence; (2) the exposure of interest was overweight or obesity defined by BMI;  (3) estimates of odds ratio (OR) or relative risk (RR) with corresponding 95% confidence intervals (CIs) (or data to calculate them) were reported. Using a case-control, cross-sectional, nested case-control, or cohort design. We placed no restriction. | 4 |
| Information sources | 7 | PubMed up to 1 February 2013  EMBASE up to 1 February 2013 | 3 |
| Search | 8 | Text words and/or Medical Subject Heading (MeSH) terms: “body mass index”, “BMI”, “overweight”, “obesity” or “excess body weight”, combined with “gallbladder cancer”, “gallbladder neoplasm” or “biliary tract cancer”  We did not employ a search software. NoteExpress was used to merge retrieved citations and eliminate duplications | 3 |
| Study selection | 9 | Details of the literature search process are outlined in the flow chart. The citation list is available upon request. We hand-searched bibliographies of retrieved papers for additional references. Two authors independently evaluated all of the studies retrieved from the databases. | 4 & Figure 1 |
| Data collection process | 10 | Three authors independently evaluated all of the studies retrieved according to the pre-specified selection criteria and extracted data using a standard data colloction form. | 4 |
| Data items | 11 | The ﬁrst author’s last name, geographic location, year of study conducted, sample size, study design, gender and age of participants, duration of follow-up (cohort studies), BMI categories, assessment of BMI (measurement versus self-reported), and the effect estimates with 95% CIs. | 4 |
| Risk of bias in individual studies | 12 | The quality of each study was assessed independently by three reviewers using the Newcastle - Ottawa Scale (NOS) | 4 |
| Summary measures | 13 | Summary relative risk (SRR) estimates with their corresponding 95%CIs were combined using a random-effects model. | 5 |
| Synthesis of results | 14 | To examine associations between overweight/obesity and the risk of GBC, we computed SRRs for two categories of BMI as defined by the [World Health Organisation (WHO)](http://www.ncbi.nlm.nih.gov/pmc/articles/PMC2360167/#bib17) for adults: overweight (BMI 25–30 kg/m^2^) and obesity (BMI > 30 kg/m^2^ or a discharge diagnosis of obesity) compared with “normal” weight (BMI 18.5–24.9 kg/m^2^). If studies reported relative risk separately for men and women, we combined the gender-speciﬁc estimates to the pool analysis. When non-standard BMI categories were provided, we selected the category that was most closed to those defined by the WHO. | 4 & 5 |

**Table S2.** *Cont.*

| **Section/topic** | **#** | | **Checklist item** | **Reported on page #** |
| --- | --- | --- | --- | --- |
| Risk of bias across studies | | 15 | Publication bias was evaluated using funnel plots and the Egger’s test. In the presence of publication bias, we used the “trim and fill” method to correct such bias. | 5 |
| Additional analyses | | 16 | To investigate the sources of heterogeneity across these studies, we carried out heterogeneity tests, and sensitivity analysis.  In heterogeneity tests, we used the Cochran Q and *I*^2^ statistics, which were used to test the differences obtained between  studies due to chance. For the *Q* statistic, a *p*-value of less than 0.10 was considered representative of statistically  significant heterogeneity. Subgroup analyses were carried out by study design (cohort and case-control studies), gender (men and women), and geographic location (non-Asia and Asia), BMI assessment (measurement and self-reported), Follow-up time (> 10 years and  < 10years), smoking status (smokers and non-smokers), Alcohol abuse (Yes and No). We conducted sensitivity analysis to estimate the influence of each individual study on the summary results by repeating the random-effects meta-analysis after omitting one study at a time. | 5 |
| **RESULTS** | | | |  |
| Study selection | | 17 | Study selection result were on page 5 & 6 combined with a flow diagram FIG1. | 5 & 6 |
| Study characteristics | | 18 | Table 1 and Table 2 |  |
| Risk of bias within studies | | 19 | Table 1 and Table 2 Column NOS |  |
| Results of individual studies | | 20 | Finally, a total of 12 cohort studies (involving 5101 cases) and 8 case-control studies (involving 1,013 cases and 43,591 controls) with data on BMI and/or obesity related to the incidence of GBC were included in the meta-analysis (Figure 1). The main characteristics of the included studies were summarized in Tables 1 and 2. 15 studies were of high quality (NOS ≥ 7). Five studies were of acceptable quality (NOS ＜ 7). | 5 |
| Synthesis of results | | 21 | As shown in Figure 2A,B, meta-analysis of all these 20 studies in a random-effects model found that a statistically significant positive association was observed between BMI and GBC risk (overweight: SRRs = 1.14, 95% CI = 1.04–1.25,  I2 = 24.9 %; obesity: SRRs = 1.56, 95% CI = 1.41–1.73, I2 = 15.4 %) compared to “normal weight”. | 6 |
| Risk of bias across studies | | 22 | No indication of publication bias was observed in the literature on BMI and GBC risk in overweight group based on the Egger’s test (*p* = 0.483) results (Figure 3A). For BMI and GBC risk in the obesity group, the funnel plot showed a little asymmetry (Figure 3B), indicating some evidence of bias. However, when the “trim and fill” approach was performed, data was unchanged, suggesting that the effect of publication bias could be negligible. | 7 |

**Table S2.** *Cont.*

| **Section/topic** | **#** | | **Checklist item** | **Reported on page #** |
| --- | --- | --- | --- | --- |
| Additional analysis | | 23 | In the sensitivity analyses, we removed one study at a time and calculating the SRRs. We found that there were no changes in the direction of effect when any one study was excluded, supporting the robustness of our results. We then conducted subgroup analyses by study design, gender, geographic location, ascertainment of exposure and adjustment for confounders, as shown in Table 3. | 7 |
| **DISCUSSION** | | | |  |
| Summary of evidence | | 24 | we found that overweight and obesity were associated with 14 % and 56 % excess risk of GBC, respectively. Our meta-analysis has several strengths. (1) This meta-analysis was based on 20 epidemiologic studies, which might minimize the possibility of selection bias. (2) Most of the included studies provided more than one RRs, which could be applicable to accurately subgroup analysis. (3) The included studies evaluated multiple confounders including smoking and alcohol. The relationships between BMI and risk of GBC in each study were derived from regression after adjustment at least for age and gender. | 8 |
| Limitations | | 25 | Our meta-analysis has limitations that affect interpretation of the true results. First, inadequate control for confounders may bias the results, leading to exaggeration or underestimation of risk estimates. Thus, when interpreting the association between excess body weight and GBC risk, possible unmeasured or residual confounding factors should be considered. Gallstone is closely related to GBC risk. Meanwhile, obesity tends to be accompanied with DM, which is also associated with increased GBC risk. However, most studies did not adjust for these risk factors. This could have led to an overestimation of the true association between obesity and risk of GBC. Second, several studies in this meta-analysis relied on self-reported weight and height data, which may attenuate the relative risk estimates. However, the SRRs for BMI ascertained by measurement were similar to those by self-reported. Finally, as in any meta-analysis, the possibility of publication bias is of concern, because a few studies with null results tend not to be published. However, the results obtained from this study did not provide evidence for such a bias. | 9 |
| Conclusions | | 26 | Findings of this meta-analysis provide evidence that excess body weight may increase GBC risk. Further studies that meet epidemiologic criteria on this subject are needed to strengthen the association between BMI and GBC risk, especially those adjusting potential confounding factors such as gallstones and DM. | 9 |

**Table S2.** *Cont.*

| **FUNDING** | | |  |
| --- | --- | --- | --- |
| Funding | 27 | This work was supported by grants from the National Natural Science Foundation of China (No. 81271876), Natural Science Foundation of Shandong Province (No. ZR2011HL004), Jining Science and Technology Project (No. 2014JNNK21-Liu Ning, 2010-Tan Wenbin), Program for Innovation of Graduate Education of Shandong Province (No. SDYY14014). | 10 |

© 2015 by the authors; licensee MDPI, Basel, Switzerland. This article is an open access article distributed under the terms and conditions of the Creative Commons Attribution license (http://creativecommons.org/licenses/by/4.0/).
